# Functional Brain Network Connectivity Patterns Associated With Normal Cognition at Old-Age, Local β-amyloid, Tau, and APOE4

**DOI:** 10.3389/fnagi.2020.00046

**Published:** 2020-03-09

**Authors:** Frances C. Quevenco, Jiri M. van Bergen, Valerie Treyer, Sandro T. Studer, Sonja M. Kagerer, Rafael Meyer, Anton F. Gietl, Philipp A. Kaufmann, Roger M. Nitsch, Christoph Hock, Paul G. Unschuld

**Affiliations:** ^1^Institute for Regenerative Medicine (IREM), University of Zurich, Zurich, Switzerland; ^2^Department of Nuclear Medicine, University of Zurich, Zurich, Switzerland; ^3^Neurimmune, Schlieren, Switzerland; ^4^Department of Psychogeriatric Medicine, Psychiatric University Hospital Zurich (PUK), Zurich, Switzerland; ^5^Zurich Neuroscience Center (ZNZ), Zurich, Switzerland

**Keywords:** MRI, functional connectivity, PET, beta-amyloid, tau, multiband fMRI, preclinical, aging

## Abstract

**Background**: Integrity of functional brain networks is closely associated with maintained cognitive performance at old age. Consistently, both carrier status of Apolipoprotein E ε4 allele (APOE4), and age-related aggregation of Alzheimer’s disease (AD) pathology result in altered brain network connectivity. The posterior cingulate and precuneus (PCP) is a node of particular interest due to its role in crucial memory processes. Moreover, the PCP is subject to the early aggregation of AD pathology. The current study aimed at characterizing brain network properties associated with unimpaired cognition in old aged adults. To determine the effects of age-related brain change and genetic risk for AD, pathological proteins β-amyloid and tau were measured by Positron-emission tomography (PET), PCP connectivity as a proxy of cognitive network integrity, and genetic risk by APOE4 carrier status.

**Methods**: Fifty-seven cognitively unimpaired old-aged adults (MMSE = 29.20 ± 1.11; 73 ± 8.32 years) were administered 11C Pittsburgh Compound B and 18F Flutemetamol PET for assessing β-amyloid, and 18F AV-1451 PET for tau. Individual functional connectivity seed maps of the PCP were obtained by resting-state multiband BOLD functional MRI at 3-Tesla for increased temporal resolution. Voxelwise correlations between functional connectivity, β-amyloid- and tau-PET were explored by Biological Parametric Mapping (BPM).

**Results**: Local β-amyloid was associated with increased connectivity in frontal and parietal regions of the brain. Tau was linked to increased connectivity in more spatially distributed clusters in frontal, parietal, occipital, temporal, and cerebellar regions. A positive interaction was observable for APOE4 carrier status and functional connectivity with brain regions characterized by increased local β-amyloid and tau tracer retention.

**Conclusions**: Our data suggest an association between spatially differing connectivity systems and local β-amyloid, and tau aggregates in cognitively normal, old-aged adults, which is moderated by APOE4. Additional longitudinal studies may determine protective connectivity patterns associated with healthy aging trajectories of AD-pathology aggregation.

## Background

Aggregation of beta-amyloid (β-amyloid) and hyperphosphorylated tau are hallmarks of brain pathology associated with Alzheimer’s Disease (AD). The age-related accumulation of both proteins precedes disease onset by decades (Price and Morris, 1999; Bateman et al., 2012). Recently published data indicate a sequential association between brain β-amyloid accumulation, subsequent tau change and resulting cognitive decline (Hanseeuw et al., 2019). However, the precise impact of β-amyloid and pathological tau on brain functionality is not yet completely understood. Several studies demonstrated the validity of positron-emission-tomography (PET) tracers for inferring the relationship between AD-pathology burden, and risk for severe cognitive impairment at old age (Klunk, 2011; Villemagne et al., 2011; Vos et al., 2013). Distinct effects on brain network connectivity have been shown for both β-amyloid and pathological tau burden (Hansson et al., 2017; Hoenig et al., 2018; Korthauer et al., 2018; Franzmeier et al., 2019; Hodgetts et al., 2019). Functional connectivity changes within the “default mode network” (DMN) relate to AD-pathology and precede the manifestation of cognitive disorder by years (Sorg et al., 2007; Sperling et al., 2009; Wang et al., 2013; Sorg and Grothe, 2015). Moreover, large clinical studies demonstrate an association between AD-pathology and brain functionality in non-demented old-aged adults, as reflected by functional network integrity (Jack et al., 2019; Maass et al., 2019; Ossenkoppele et al., 2019). This may be consistent with functional network change in other neurodegenerative diseases (Rektorova et al., 2012; Ross et al., 2014; Kronenbuerger et al., 2019).

Despite the consensus in the literature that AD-pathology is related to a breakdown in functional brain networks, its associations with functional connectivity are tenuous. The relationship between β-amyloid and decreased connectivity, mainly in the DMN, is well established for clinically manifest AD (Hedden et al., 2009; Sheline et al., 2010; Chhatwal et al., 2013; Wang et al., 2013). However, during aging and in the absence of cognitive disorder, both increased functional connectivity (Mormino et al., 2011; Lim et al., 2014), but also decreased network connectivity (Wang et al., 2013) has been observed to be associated with AD-pathology when assessing total β-amyloid load, and tau burden.

The posterior cingulate and precuneus (PCP) represents a central node of the DMN needed for memory processing (Sperling et al., 2009; Cieri and Esposito, 2018). β-amyloid load affects the functionality of the PCP in old aged adults with, and without cognitive impairment (Unschuld et al., 2012; Schreiner et al., 2016; Grothe et al., 2017; Quevenco et al., 2017, 2019). The Apolipoprotein E ε4 allele (APOE4) is the strongest known genetic risk factor for late-onset AD (Corder et al., 1993; Strittmatter et al., 1993; Beffert and Poirier, 1996). In the general population, APOE4 carrier status is associated with an increased risk for the presence of AD-pathology (Schipper, 2011; Verghese et al., 2011; Liu et al., 2013; Jack et al., 2019; Yamazaki et al., 2019). Consistently, brain network alterations involving the PCP are pronounced in carriers of the APOE4 genotype (Sperling et al., 2009; De Vogelaere et al., 2012).

PET using tracers 18F-Flutemetamol and Pittsburgh Compound B (PiB) by now is a well-established neuroimaging technique for measuring individual β-amyloid plaque burden (Vandenberghe et al., 2010; Klunk, 2011; Frisoni and Blennow, 2013). Moreover, PET tau tracers such as 18F AV-1451 have enabled *in vivo* assessment of pathological, hyperphosphorylated tau burden in humans (Schöll et al., 2017; Mainta et al., 2018; Villemagne et al., 2018). These advances in PET technologies allow for investigating the relationship of β-amyloid and tau, whose interactions are considered to significantly contribute to disease progression and functional decline (Selkoe and Hardy, 2016; Tosun et al., 2017; Kametani and Hasegawa, 2018) Spatial distribution patterns of both proteins diverge substantially, with β-amyloid accumulation beginning in the neocortex and progressing towards subcortical structures (Braak and Braak, 1991a), whereas tau accumulation begins in the brainstem and transentorhinal regions and spreads towards neocortical structures as the disease progresses (Braak and Braak, 1991b). Considering these earlier reports, we hypothesized that PCP functional connectivity in cognitively normal old-aged adults might reflect the interplay between β-amyloid and tau pathology on a local level. Moreover, we hypothesized, that such effects should be more pronounced in APOE4 carriers. To test these hypotheses, the relationship between local β-amyloid, local tau and PCP connectivity was investigated on a voxel-level. We used the software biological parametric mapping (BPM; Casanova et al., 2007) for generating subject-specific PET tracer retention patterns and beta-maps (Whitfield-Gabrieli and Nieto-Castanon, 2012) as a measure of individual connectivity patterns (Biswal et al., 1995), associated with AD pathology. High temporal resolution of connectivity data was achieved by using multiband functional MRI (fMRI) for the simultaneous acquisition of multiple slices (Feinberg and Setsompop, 2013; Preibisch et al., 2015).

## Materials and Methods

### Study Population

For the current study, 57 old-aged cognitively unimpaired (mean MMSE = 29.20 ± 1.11), and medically healthy adults (mean age = 73 ± 8.32 years) were included from an on-going neuroimaging study at our institute, using study procedures reported earlier (van Bergen et al., 2018a; Vandenberghe et al., 2010). Written informed consent was obtained from all participants before inclusion in the study. Study procedures were carried out in accordance with the Good Clinical Practice (GCP) guidelines issued by the local ethics authority (Kantonale Ethikkommision Zürich^1^), and with the Human Research Act of Switzerland and the declaration of Helsinki (World_Medical_Association, 1991).

Inclusion criteria were age above 50 years, pre-existing PET information on cerebral amyloid deposition, German language proficiency and capacity to consent to study procedures. Exclusion criteria were: significant medication or drug abuse that might have possible effects on cognition, a history of severe allergic reaction or known allergy against components of 11C-PiB, 18-Flutemetamol, AV-1451, general MRI exclusion criteria, MRI scans with evidence of infection or infarction and severe atrophy, clinically relevant changes in red blood cell count, serious medical or neuropsychiatric illness and significant exposure to radiation.

### Flutemetamol and PiB-PET for Estimation of β-amyloid Load

Individual measures of local β-amyloid burden were determined by quantification of standardized uptake values of either 18F-Flutemetamol (34 participants) or 11C-Pittsburgh Compound B (11C-PiB, 23 participants). PET was performed as reported in earlier studies of ours (Schreiner et al., 2014; Steininger et al., 2014; van Bergen et al., 2018a). A General Electric (GE) healthcare SIGNA PETMR was used for measuring 18F-Flutemetamol uptake, and a GE healthcare Discovery scanner for 11C-PiB. Average and standard deviation (SD) maps of all amyloid-PET SUVR are indicated in Supplementary Figures S1, S2. For the former, an individual dose of approximately 140 MBq of Flutemetamol was injected into the cubital vein. Time-of-flight algorithms and the required corrections were applied to reconstruct the PET images. Late-frame values (85–105 min) were referenced to the cerebellar gray matter (Vandenberghe et al., 2010) to obtain 3D volumes of Flutemetamol retention as an estimate β-amyloid load using standardized uptake value ratios (SUVR; matrix = 256 × 256 × 89, voxel size = 1.2 × 1.2 × 2.78 mm^3^). For the PiB protocol, approximately 350 MBq were administered intravenously and late-frame values (50–70 min) were used to obtain mean PiB uptake in all cortical VOIs and cerebellar regions using a volume-weighted averaging procedure (Gietl et al., 2015). Identical to the Flutemetamol protocol, these were normalized and referenced to the cerebellar gray matter to obtain SUVRs as estimates of the β-amyloid load.

### AV1451-PET for the Estimation of Tau Load

The current study also included measures of tau load, by assessing local retention of 18F-AV1451 using an established PET procedure (Chien et al., 2013). A GE healthcare SIGNA PETMR was used for measuring 18F-AV1451 uptake. Average and SD maps of all tau-PET SUVR are indicated in Supplementary Figures S3, S4. In our study, an individual dose of approximately 200 MBq of 18F-AV1451 was administered into the cubital vein. Similar to the β-amyloid procedure, time-of-flight algorithms with the necessary corrections were applied to construct the tau-PET images based on regional uptake values. For analysis, 8 × 5 min frames were acquired. These were summed to late-frame values (80–120 min) and referenced to the cerebellar gray matter for generation of SUVRs.

### MRI Acquisition

MR-Images were acquired on a 3-Tesla GE Discovery 750w MR whole-body scanner (GE Medical Systems, Milwaukee, WI, USA) equipped with a 32 Channel head coil array. A 3D T1-weighted (IR-SPGR) sequence (TI = 600 ms, TE = minimum, voxel size = 1 × 1 × 1 mm^3^, FOV = 256 × 256 × 256 mm^3^, flip angle = 8°, scan time = 5:49 min) was used to obtain structural images for anatomical referencing and automated image segmentation at the same visit the AV1451-PET was performed for tau. The average time between MRI and β-amyloid PET was 1,138 days, with a minimum of 49 days, and a maximum of 2,414 days.

Resting-state BOLD fMRI images were acquired using a 2D Gradient Echo Mux Multiband sequence (TR = 2,000 ms, TE = 30 ms, flip angle = 70°, slice thickness = 2.4 mm, number of slices = 22, number of MUXed slices (1–8) = 3, Autocalibrating Reconstruction for Cartesian imaging (ARC) acceleration factor = 2, ARC enabling = 1, scan time = 8:32 min) to obtain individual beta maps, indicating functional connectedness of each voxel to the PCP based on BOLD synchronicity (Biswal et al., 1995; Whitfield-Gabrieli and Nieto-Castanon, 2012). Average and SD maps PCC connectivity are indicated in Supplementary Figures S5, S6.

### Pre-processing of Functional Images and Obtaining PCP Seed Maps

Functional images were spatially preprocessed using an in-house script implementing functions from the SPM12 toolbox^2^ with MATLAB 2016a (Mathworks, Natick, MA, USA). The following spatial preprocessing measures were covered in the script: (1) Functional image time-series were realigned using a 6-parameter rigid-body transformation for each image. (2) Anatomical (T1) scans were coregistered to the mean functional image. (3) Structural T1 images were segmented using SPM12’s Segment function in order to obtain tissue probability maps to warp the functional images into normalized MNI space. (4) Functional and structural images were normalized by using the forward deformations obtained in the previous step to warp the images from native into MNI space. (5) Normalized functional images were then smoothed with an 8 mm FWHM Gaussian kernel. Slice-time correction is not a necessity with multiband images due to the low TR, which reduces temporal differences between slices. In addition, as multiband acquires a high number of slices, the slight movement could have corrections done for the wrong slice. In order to avoid this complication, this step was omitted from spatial preprocessing. Normalized and smoothed functional and normalized T1 images were processed using the CONN v12 toolbox (Whitfield-Gabrieli and Nieto-Castanon, 2012). This toolbox uses the CompCor denoising method procedure, which regresses out nuisance regressors without including the global signal. After the denoising step, PCP seed maps were obtained using the CONN toolbox by calculating the correlation between the PCP and other regions of the brain.

### Biological Parametric Mapping (BPM) for Voxel-Level Analysis

**Figure 1:**
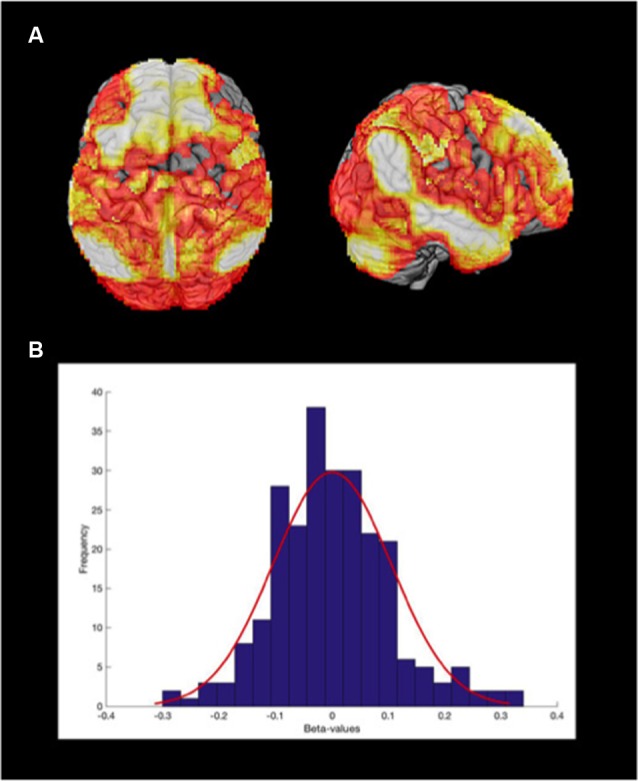
Posterior cingulate and precuneus (PCP) connectivity seed map of sample population. **(A)** Connectivity map of the whole sample (*p-FDR* < 0.05), using the PCP as a seed, projected on a 3D brain. **(B)** The median distribution of the Fisher r-to-z transformed beta maps.

PET images were, based on their co-registered T1 image, aligned to the TAU PET/MR T1 image using the PMOD software, Version 3.8 (PMOD Technologies Limited, Zurich, Switzerland). T1 MRI images were normalized to MNI space and matrices were applied to the PET images, which were then scaled by cerebellum gray matter values derived from an intersected Hammers atlas derived gray matter region, which fit all normalized PET images. These were then smoothed with a 5 mm FWHM Gaussian kernel. Preprocessing of the functional images for BPM analysis required realignment, coregistration, segmentation and warping into MNI space and finally smoothed with an 8 mm FWHM Gaussian kernel using in-house scripts running on SPM12\footnotemark with MATLAB 2016a (Mathworks, Natick, MA, USA). The smoothed and normalized functional images were processed using the CONN v.12 toolbox (Whitfield-Gabrieli and Nieto-Castanon, 2012). Individual Fisher r-to-z transformed beta PCP seed connectivity maps were calculated for each participant. A second level *t*-test against zero was applied on the data and multiple test correction was performed at the voxel level, using FDR (*p-FDR corrected, q* < 0.05 for p-uncorrected = 0.001, Figure 1). To limit the analysis to the gray matter, images were masked using a multi-atlas matching approach (van Bergen et al., 2018b). Regression analysis of normalized and smoothed PET and fMRI images was performed by administering BPM^3^ (Casanova et al., 2007; Yang et al., 2011), a toolbox useful for exploring multimodal voxel-level correlations using the General Linear Model (GLM), running under Matlab 2010a and SPM5^4^. The common spatial resolution of the images used in the analysis was determined by the spatial modality of PET (the lowest resolution) of approximately 3 mm. BPM was used to investigate regression models for investigating the relationship between local β-amyloid PET, tau-PET and DMN connectivity, as measured by beta maps, with a BPM cluster threshold set at 100 voxels. Additionally, the potential moderating effects of APOE4 carrier status were explored by multiple regression.

## Results

### Neuropsychological Performance and APOE4 Carrier Frequency

All participants underwent a battery of neuropsychological tests including the Mini-Mental State Exam (MMSE), the Boston Naming, Trail-Making and the VLMT Delayed Recall test. Mini-Mental State (MMSE) scores were within the normal range (mean MMSE = 29.20 ± 1.11), and the other neuropsychological tests indicated consistently high performance of the studied population in the investigated domains (Supplementary Table S1). Moreover, genotyping identified 11 APOE4 carriers within the 57 study participants (19.3% of the population).

### Local β-amyloid and Tau Deposits Are Associated With Locally Increased Functional Connectivity

**Figure 2:**
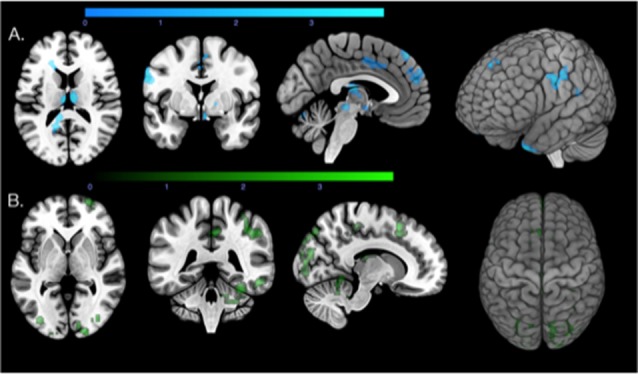
Regions that show correlations between local β-amyloid, local tau, and increased functional connectivity. **(A)** Axial, coronal, and sagittal slices and a 3D render of the T-map (*p* < 0.05, extent threshold = 100 voxels) illustrating the regions with correlations between local β-amyloid and increased connectivity (blue). **(B)** Axial, coronal, and sagittal slices and a 3D render of the T-map (*p* < 0.05, extent threshold = 100) illustrating the regions with correlations between local tau load (green) and increased functional connectivity.

By applying voxel-wise regression, eight positively associated clusters with a size of at least 100 voxels (Total voxels = 2,030, *T* > 2.3, *p* < 0.05) could be identified, indicating relationships between local β-amyloid load and increased PCP connectivity. Clusters were mainly located in the frontal and parietal lobe (Figure 2A), including the middle frontal gyrus, the anterior orbital gyrus (right), the medial orbital gyrus (right), the lateral orbital gyrus (right), the left precentral gyrus, the inferior lateral remainder of the parietal lobe (right) and other regions such as the left anterior temporal lobe (medial part) and the left cerebellum. No significant association between local β-amyloid burden and decreased PCP connectivity could be observed. Also, when applying voxel-wise regression, no significant relationship could be observed between age and gender, and PCP connectivity.

For local tau associated PCP connectivity changes, the voxel-based analysis identified 14 positively associated clusters with a minimum size of 100 voxels (Total voxels = 5,662, *T* > 2.26, *p* < 0.05) widely distributed across the brain (Figure 2B). Regions primarily involved were thalamus, superior frontal gyrus, middle frontal gyrus, the inferolateral remainder of the parietal lobe (right), superior parietal gyrus, the lateral remainder of the occipital lobe, posterior temporal lobe and middle and inferior temporal gyrus. Smaller clusters included the cingulate gyrus and the right cerebellum.

**Figure 3:**
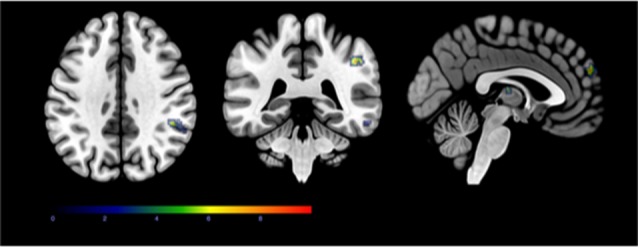
The minor regional overlap between local β-amyloid and increased PCP connectivity and local tau and increased PCP connectivity. Axial, coronal, and sagittal slices showing the small regional overlap of regions that are associated with local β-amyloid and increased PCP connectivity (*p* < 0.05, extent threshold = 100 voxels), and local tau-load and increased PCP connectivity (*p* < 0.05, extent threshold = 100 voxels).

There were small regions of overlap (257 voxels) between local β-amyloid associated connectivity and tau-related connectivity (Figure 3), representing ca. 12.66% of voxels included in the β-amyloid PCP connectivity map and 4.54% of the tau PCP connectivity map. The overlap between β-amyloid and tau-related connectivity was observable in particular for the left and right cerebellum, superior frontal gyrus and the inferolateral remainder of the parietal lobe (right).

### APOE4 Carrier Status Moderates the Association Between β-amyloid and Tau Load With Functional Connectivity

**Figure 4:**
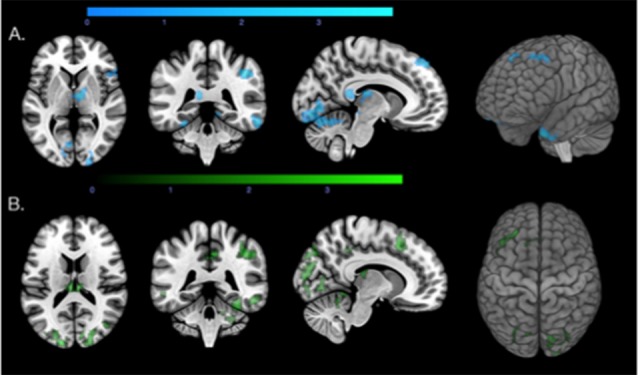
Regions with increased connectivity associated with local β-amyloid, local tau, and Apolipoprotein E ε4 allele (APOE4) carrier status. **(A)** Axial, coronal, and sagittal slices and a 3D view of the biological parametric mapping (BPM) T-map (*p* < 0.05, extent threshold = 100) illustrating regions with associations between local β-amyloid, increased functional connectivity, moderated by APOE4 carrier status (blue). **(B)** Axial, coronal, and sagittal slices and a 3D view of the BPM T-map (*p* < 0.05, extent threshold = 100) illustrating regions that showed associations between local tau, increased connectivity, moderated by APOE4 carrier status (green).

**Figure 5:**
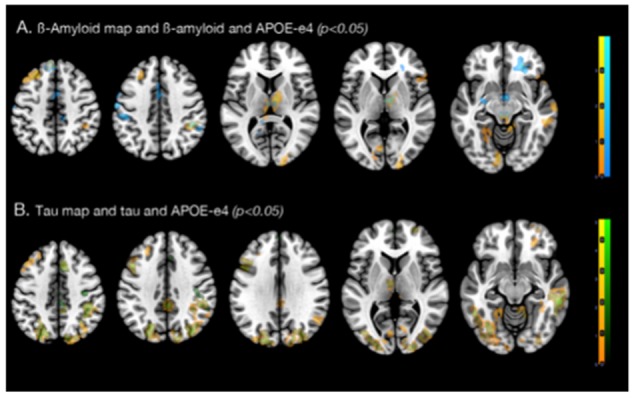
Effects of APOE4 carrier status on regional associations between increased connectivity with **(A)** local β-amyloid, and **(B)** local tau. **(A)** Axial slices of regions with correlations between local β-amyloid and increased connectivity (blue), and local β-amyloid, APOE4 carrier status and increased PCP connectivity (orange). **(B)** Axial slices of regions associated with local tau-load and the Fisher r-to-z transformed beta-maps (green), local tau-load, APOE4 carrier status and increased PCP connectivity (orange).

APOE4 carrier status was included in the regression model as a non-imaging covariate and was found to have positive moderator effects on both β-amyloid and tau-related functional connectivity properties (Figure 4). APOE4 carrier status and local β-amyloid load were associated with increased PCP connectivity in 14 clusters of at least 100 voxels (Total voxels = 3,666, *T* > 2.80, *p* < 0.05). In contrast to the regression model with just β-amyloid and the Fisher r-to-z beta maps, which found regions with the strongest associations in the frontal lobe, APOE4 status and local β-amyloid were associated with more widely distributed PCP connectivity changes primarily in the temporal and frontal lobe (Figure 5A). These included the left anterior temporal lobe (medial and lateral part), posterior temporal lobe, right middle and inferior temporal gyrus, left middle frontal gyrus, left precentral gyrus, right medial orbital gyrus, and the right anterior orbital gyrus. Other regions included in the clusters were the right thalamus, the inferolateral remainder of the parietal lobe and the left lingual gyrus in the occipital lobe.

Local tau-tracer retention and APOE4 carrier status were associated with increased PCP connectivity in 13 clusters with at least 100 voxels in size (Total voxels = 7,045, *T* > 2.5, *p* < 0.05). Similar to the findings from the local tau and connectivity regression model, regions with strong associations between increased connectivity, local tau, and APOE4 carrier status were widely distributed across the brain (Figure 5B). Primary regions were located in the neocortex and also include other central structures, such as the cerebellum, the thalamus and caudate nucleus. These clusters enclosed brain regions within the right superior frontal gyrus, middle frontal gyrus, superior parietal gyrus, the lateral remainder of the occipital lobe, right middle and inferior temporal gyrus, and posterior temporal lobe. Similarly as with β-amyloid, no significant association between local retention of the tau-tracer and decreased PCP connectivity could be observed.

## Discussion

By applying a multimodal voxel-wise regression approach to non-demented old-aged adults, we identified relationships between local β-amyloid and tau deposits with functional connectivity changes of the PCP. While local aggregation of both proteins was associated with distinct patterns of functional hyperconnectivity, the regional overlap was small. As we did find a positive moderator effect of APOE4 for both β-amyloid and tau related connectivity, we assume potential relevance for risk and progression of AD.

The majority of studies in the past have found decreased functional connectivity in the presence of AD (Sheline et al., 2010; Ouchi and Kikuchi, 2012; Song et al., 2013) and β-amyloid deposition (Sperling et al., 2010), particularly affecting the DMN and even reduction of connectivity in specific DMN nodes, such as the posterior cingulate (Weiler et al., 2014). There is however a handful of contradicting findings that observed increased connectivity and postulated that these changes may be exemplary of early-stage compensatory mechanisms in reaction to brain pathology (Mormino et al., 2011; Lim et al., 2014).

Though the reduction in connectivity is a prominent finding in the literature, our results coincide with a longitudinal PET connectivity study (Jack et al., 2013) and a recent β-amyloid and tau study (Schultz et al., 2017), that suggests that along the AD spectrum there are phases of hyper- and hypoconnectivity, with the former preceding the latter. Moreover, our findings are consistent with recent data on functional alterations associated with the presence of AD-pathology in non-demented old-aged adults (Jack et al., 2019; Maass et al., 2019; Ossenkoppele et al., 2019). Considering that the here investigated study population still had a low β-amyloid burden (*A*β−) and was cognitively relatively healthy, the observed increase in PCP connectivity may reflect the presence of pathological impairment prior to significant β-amyloid aggregation. The observed β-amyloid associated PCP connectivity was focal and significant clusters were located in the frontal and temporal lobe. Tau, however, was associated with increased activity in a wider distribution of regions across the brain. Only limited spatial overlap between PCP connectivity networks affected by β-amyloid and tau were observable. Overlap was present in the superior frontal gyrus, the cerebellum and the inferolateral remainder of the parietal lobe. While our data needs to be interpreted with caution due to the low level of PET tracer retention in an *A*β− population, possible off-target tau signal (Baker et al., 2019) and the possibility of slightly different attenuation correction due to the use of different PET scanners, our data nevertheless may accord with recent fMRI studies that suggest distinct PCP functional connectivity networks associated with β-amyloid and tau (Sepulcre et al., 2017; Franzmeier et al., 2019).

Interestingly, we found more clusters to be associated with local β-amyloid load and APOE4 carrier status than β-amyloid alone and these clusters are no longer primarily in the frontal lobe but cover many parts of the neocortex as well as other central structures, such as the cerebellum, thalamus and the caudate nucleus. While the addition of the genetic AD risk factor to the regression model better explains PCP associated increased connectivity in our data, this may be consistent with earlier reports on APOE4 associated connectivity changes in non-demented populations (Westlye et al., 2011; Wang et al., 2015; Hodgetts et al., 2019). However, this relationship was less prominent for local tau and PCP connectivity. Although adding APOE4 carrier status as an independent covariate did show some regions that were not affected with only tau alone, APOE4 effects on tau appeared less widespread. While our findings might support earlier reports on an association between APOE4 and tauopathy (Beffert and Poirier, 1996; Tiraboschi et al., 2004; Shi et al., 2017), in our study APOE4 moderated the relationship between local tau and functional connectivity to a lesser degree than β-amyloid.

Nonetheless, this observation could be a reflection of the relatively healthy status of our population, where neurodegenerative brain damage, indicated by increased tau, may still be very limited, resulting in low power for the detection of interactive effects between tau, APOE4 and functional connectivity. This may accord with the amyloid cascade hypothesis, which postulates that β-amyloid dysregulation precedes tau related neurodegeneration in preclinical stages of AD (Hardy and Selkoe, 2002). Furthermore, this study only finds relationships between β-amyloid and tau with increased PCP connectivity. Our finding of distinct functional impacts of β-amyloid and tau might be consistent with the notion of pathogenic synergism of protein aggregates in aging and neurodegenerative disease (Nelson et al., 2012). However, our findings may also reflect other non-AD related tauopathy, which might concur with very early stages of potential β-amyloid pathology in the studied population. Possible implications for therapeutic intervention strategies against age-related cognitive decline have been reviewed recently (Pini et al., 2018).

For the current study multiband fMRI was used, allowing for increased temporal resolution of resting-state data (Feinberg and Setsompop, 2013; Preibisch et al., 2015) while maintaining reproducibility of results with conventional EPI based fMRI protocols (Smitha et al., 2018). The increase in temporal resolution made possible by multiband fMRI has been suggested to allow for novel insights in brain network dynamics (Preibisch et al., 2015).

However, the cross-sectional nature and small sample size is a limitation of our study. Furthermore, due to the small sample size, further stratification of the studied population by AD-relevant properties is not compatible with the chosen statistical analysis strategy. While we did not find effects of gender and age, these might be present in a larger study population, particularly if a wider age range is included. Also, the specific investigation of APOE4 carriers, and also limiting the analysis to individuals with high cerebral β-amyloid (*A*β+ vs. *A*β−), and effects associated with tau progression beyond the entorhinal cortex, may provide additional insights on AD pathology (Braak and Braak, 1991b; Thal et al., 2002). While it is difficult to conclude whether our findings reflect pathological stages associated with either β-amyloid or tau, recent data suggest a sequential association of pathological change in AD (Hanseeuw et al., 2019). Prospective, longitudinal studies may include trajectories of gray matter integrity, as demonstrated recently for estimating neural tissue disintegration (Koini et al., 2018). Moreover, as we investigated a relatively healthy population, information on a relationship between connectivity-properties and structural change of vulnerable brain regions is limited. At present, 18F AV-1451 has become a popular choice for *in vivo* tau imaging (Villemagne et al., 2018; Mattsson et al., 2019; Smith et al., 2019). However, AV-1451 off-target binding unrelated to tau is a frequent finding particularly at low tracer retention, as observed in our study. Here, AV-1451 off-target signal may reflect iron deposition, neuromelanin, or vascular factors (Lockhart et al., 2017). This particularly affects brain regions such as the thalamus, putamen, superior cerebellar gray, choroid plexus, but also meninges and skull, posing challenges when studying early tau accumulation, as done in the current study (Baker et al., 2019). Functional coupling between the PCP and the PCC is well known, and our finding of an association with local β-amyloid may accord with earlier reports on altered functional brain changes during aging (Steininger et al., 2014; Li et al., 2017; Liu et al., 2017; Luo et al., 2019). However, considering that no significant cerebellar β-amyloid accumulation is to be expected in cognitively healthy individuals (Thal et al., 2002), also this finding needs further replication, and for now, needs to be interpreted with caution. Another limitation is the fact that there was significant temporal delay between β-amyloid PET and MRI, while tau PET and MRI took place at once. This difference needs to be taken into account when interpreting our findings on connectivity effects associated with β-amyloid vs. tau. However, β-amyloid effects may manifest in the long term, considering that aggregation of β-amyloid has been estimated to take about 19 years from β-amyloid positivity-threshold to mean values observed in AD dementia (Roberts et al., 2017).

Moreover, although the PCP is considered to play a central role in various cognitive processes associated with the DMN (Choo et al., 2010; Teipel et al., 2015; Quevenco et al., 2017; Cieri and Esposito, 2018), PCP seed map-related effects observed in this study may nevertheless primarily reflect PCP specific pathology than general alterations of the DMN. Follow-up studies using whole-brain connectivity approaches both at rest and during cognitive tasks may thus expand findings of the current study to investigate the DMN and also other brain networks in the context of AD-risk. Here, the advent of novel blood-based biomarkers might help to further characterize processes resulting in impaired brain functionality in preclinical AD (Bacioglu et al., 2016; Mattsson et al., 2017; Zetterberg and Blennow, 2018).

In conclusion, our findings are consistent with recently published studies that describe phases of PCP hyperconnectivity during the early stages of age-related β-amyloid and tau-related aggregation (Sepulcre et al., 2017). Considering the central role of the PCP for DMN connectivity, our study suggests that APOE4 carrier status, aggregation of β-amyloid and tau closely interact regarding their effects on DMN connectivity. Additional studies are needed to clarify connectivity in the context of the sequential progression of AD pathology (Hanseeuw et al., 2019), early functional change (McLaren et al., 2012), and potential opportunities for therapeutic intervention (Pini et al., 2018). The fact that these effects are observable before the manifestation of dementia, but still are related to APOE4 carrier status, may indicate relevance for the progression of early AD-pathology (Haller et al., 2019). Thus, further longitudinal studies are needed to investigate the temporal sequence of the observed changes, and whether they are associated with accelerated disease progression. Such studies may also inform on functional connectivity in a context of resilience against the aging-related accumulation of AD pathology. Possibly protective effects of functional connectivity patterns may allow for compensating emerging brain pathology during aging, allowing for preserved cognition.

## Data Availability Statement

Openly available datasets generated for this study are included in the article/Supplementary Material.

## Ethics Statement

The studies involving human participants were reviewed and approved by Kantonale Ethikkommission Zürich. The patients/participants provided their written informed consent to participate in this study.

## Author Contributions

FQ: performed data processing, analysis and interpretation of neuroimaging data and contributed to writing of the manuscript together with PU, and has performed a final revision. JB: established the multiband fMRI protocol on the GE scanner in Schlieren, and installed the pipeline for performing multimodal analyses using Biological Parametric Mapping. Also, he performed data processing of MR- and PET-data for integrated, voxel-level analysis. SS, AG, RM, and VT: contacted participants, administered MRI and PET. AG: coordinated the study and genotyping of APOE, interaction with ethics committee and acquisition of PET-MR data. SK: revised MR-data analysis and wrote the manuscript. RM and VT: supervised data acquisition (MRI and PET). VT and PK: supervised the preparation of the 18F-tracer for measuring brain Aβ-plaque density, quality control of PET-data and analysis. RN and CH are the chairmen of the department and sponsors of the study, they provided critical revisions of the final manuscript. PU: proposed the research question, supervised all data analysis, wrote the manuscript and is corresponding author. Also, he supervised the acquisition, processing, analysis, interpretation of acquired data and has performed the final revision.

## Conflict of Interest

RN (CEO) and CH (CMO) are members of the board of directors at Neurimmune, Schlieren, Switzerland. The remaining authors declare that the research was conducted in the absence of any commercial or financial relationships that could be construed as a potential conflict of interest.

## Footnotes

^1^www.kek.zh.ch

^2^http://www.fil.ion.ucl.ac.uk/spm/software/spm12

^3^https://www.nitrc.org/projects/rbpm/

^4^https://www.fil.ion.ucl.ac.uk/spm/software/spm5/

## Supplementary Material

FIGURE S1 Amyloid average over all PET scans performed (18F Flutemetamol and 11C PiB). Scaled from 0 to 2.8 SUVR.

FIGURE S2 Amyloid standard deviation (SD) over all PET scans performed (18F Flutemetamol and 11C PiB). Scaled from 0 to 0.8 SUVR SD.

FIGURE S3 Tau average over all PET scans performed (18F AV1451). Scaled from 1.0 to 2.0 SUVR.

FIGURE S4 Tau SD over all PET scans performed (18F AV1451). Scaled from 0 to 0.6 SUVR SD.

FIGURE S5 Beta-maps average, scaled from 0 to 1.0.

FIGURE S6 Beta-maps SD, scaled from 0 to 0.3.
