# Supplementary figures and images for: Functional Brain Network Connectivity Patterns Associated With Normal Cognition at Old-Age, Local β-amyloid, Tau, and APOE4
Source: Front Aging Neurosci. 2020 Mar 9;12:46. doi: 10.3389/fnagi.2020.00046 (PMC7075450; doi:10.3389/fnagi.2020.00046)

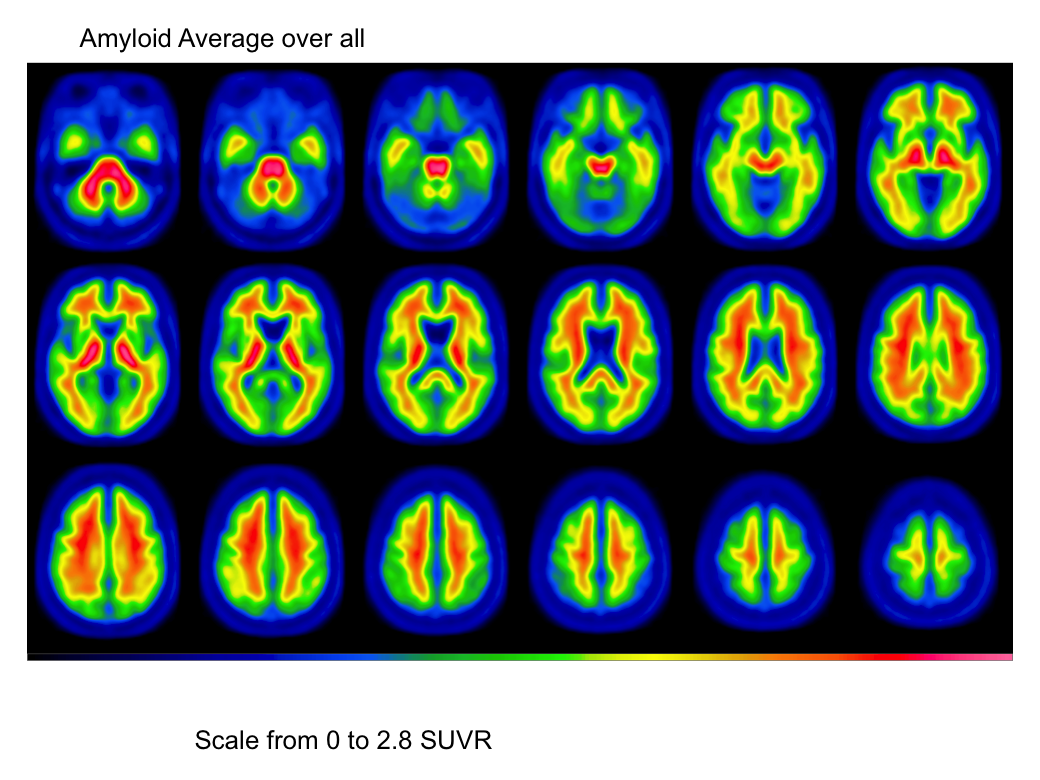

Supplement: FIGURE S1 — Amyloid average over all PET scans performed (18F Flutemetamol and 11C PiB). Scaled from 0 to 2.8 SUVR. [file Image_1.tiff]

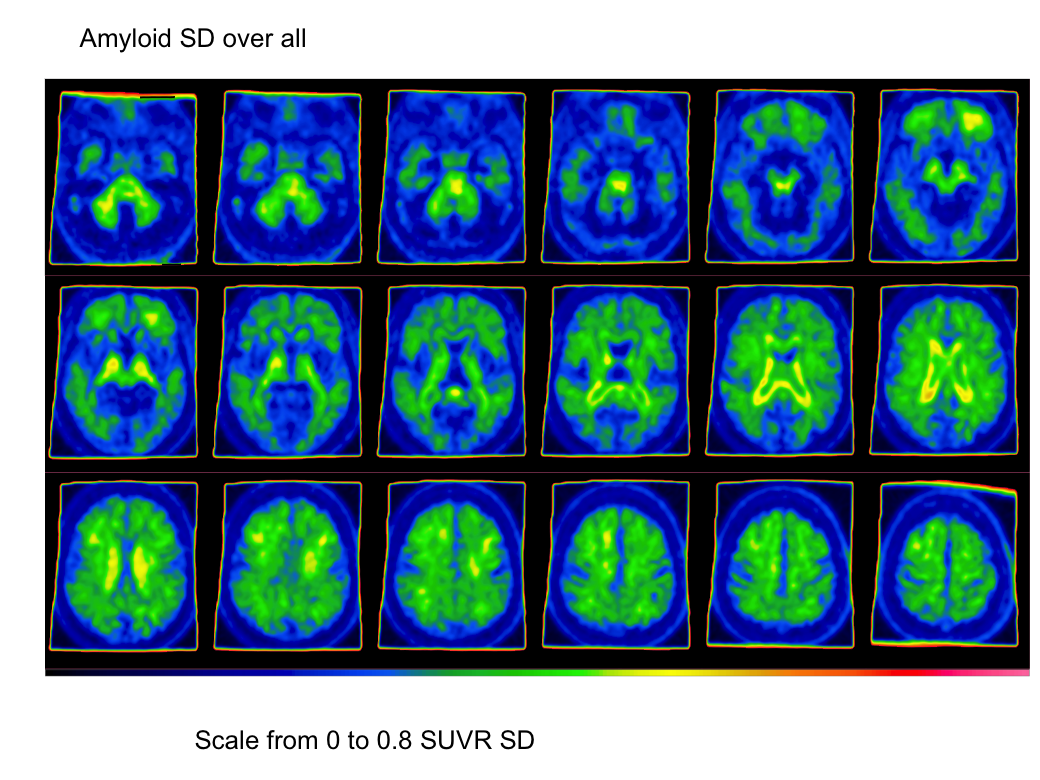

Supplement: FIGURE S2 — Amyloid standard deviation (SD) over all PET scans performed (18F Flutemetamol and 11C PiB). Scaled from 0 to 0.8 SUVR SD. [file Image_2.tiff]

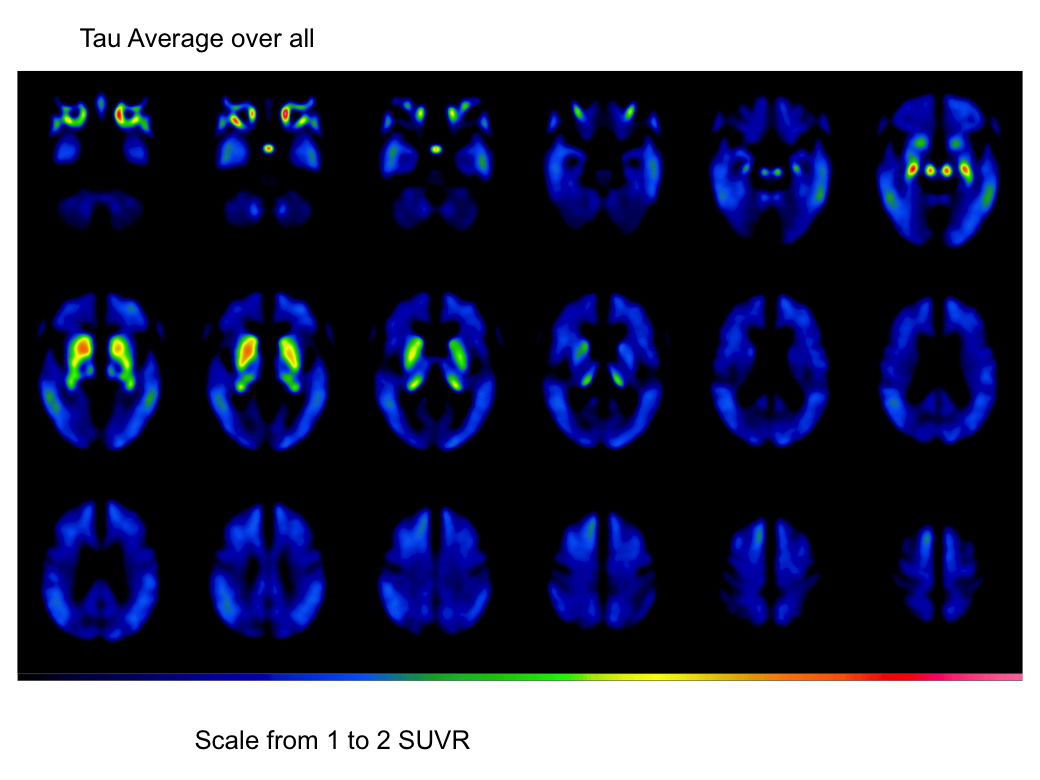

Supplement: FIGURE S3 — Tau average over all PET scans performed (18F AV1451). Scaled from 1.0 to 2.0 SUVR. [file Image_3.tiff]

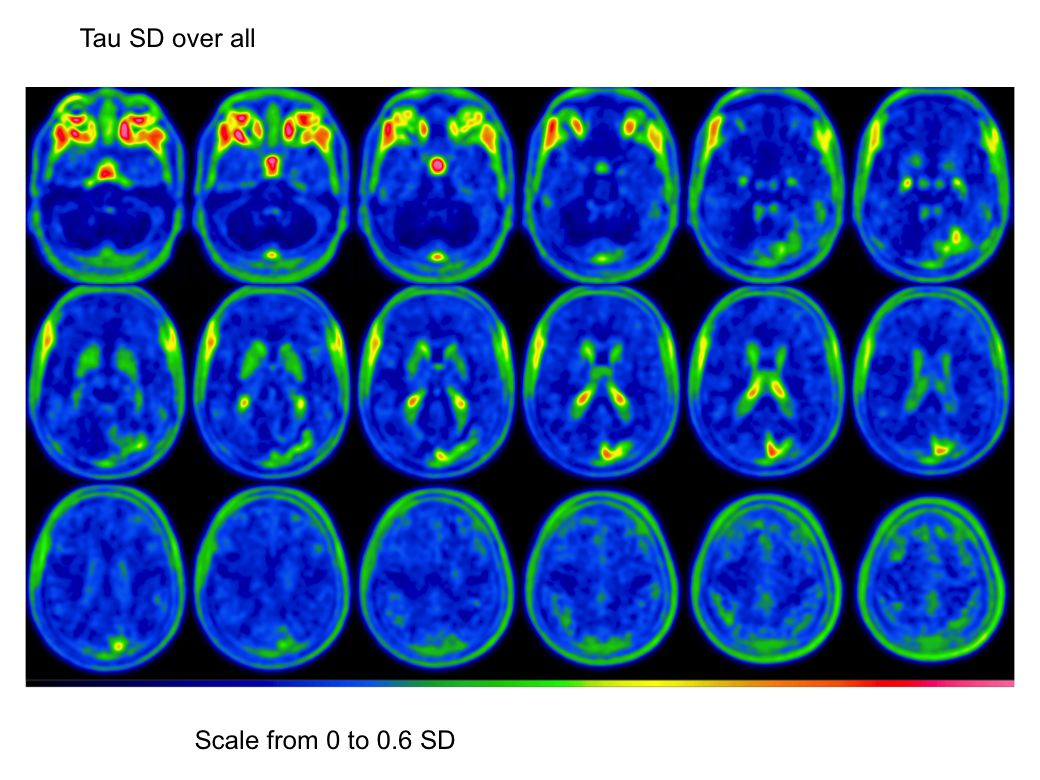

Supplement: FIGURE S4 — Tau SD over all PET scans performed (18F AV1451). Scaled from 0 to 0.6 SUVR SD. [file Image_4.tiff]

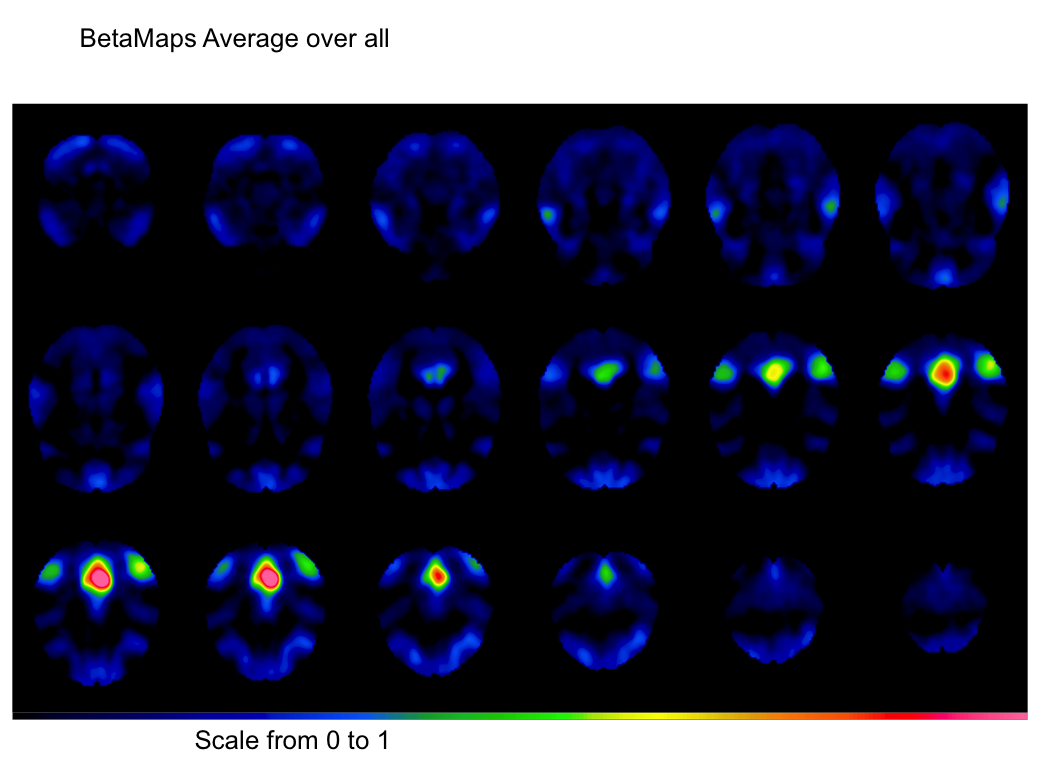

Supplement: FIGURE S5 — Beta-maps average, scaled from 0 to 1.0. [file Image_5.tiff]

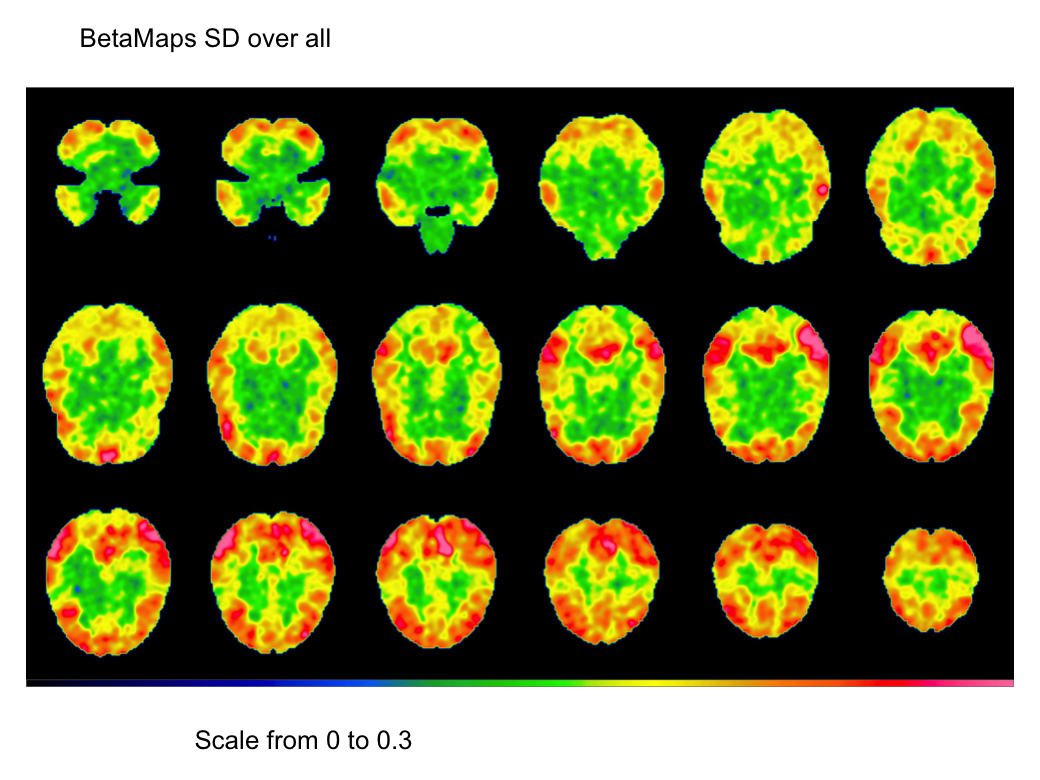

Supplement: FIGURE S6 — Beta-maps SD, scaled from 0 to 0.3. [file Image_6.tiff]
