# Supplementary material for: Functional Brain Network Connectivity Patterns Associated With Normal Cognition at Old-Age, Local β-amyloid, Tau, and APOE4
Source: Front Aging Neurosci. 2020 Mar 9;12:46. doi: 10.3389/fnagi.2020.00046 (PMC7075450; doi:10.3389/fnagi.2020.00046)
Supplement: Supplementary file 7 [file Table_1.DOCX]

***Table 1.*** Demographics and neuropsychological test performance.

| **Demographics** |  |
| --- | --- |
| Age (years) | 73±*8.32* |
| N (females ; males) | 57 (19; 38) |
| Formal education (years) | 16.19±*3.03* |
| BMI | 25.08±*3.13* |
| APOE4 carriers (n) | 11 |
|  |  |
| **Neuropsychology, test performance** |  |
| MMSE | 29.20±*1.11* |
| Boston Naming Test | 14.65±*0.67* |
| Digit Span Forward | 7.05±*1.58* |
| Digit Span Backward | 6.30±*1.43* |
| Trail-Making Test | 2.50±*0.72* |
| VLMT Delayed Recall | 9.67±*4.40* |
